# Application of Fine-Grain Carbon Materials in the Process of Smelting Lead from Battery Paste

**DOI:** 10.3390/ma17235806

**Published:** 2024-11-27

**Authors:** Grzegorz Siwiec, Tomasz Matula, Magdalena Kokowska-Pawłowska

**Affiliations:** 1 Department of Metallurgy and Recycling, Faculty of Materials Science, Silesian University of Technology, Krasinskiego 8, 40-019 Katowice, Poland; grzegorz.siwiec@polsl.pl; 2 Department of Applied Geology, Faculty of Mining, Safety Engineering and Industrial Automation, Silesian University of Technology, Akademicka 2, 44-100 Gliwice, Poland; magdalena.kokowska-pawlowska@polsl.pl

**Keywords:** lead–acid batteries, pyrometallurgy, carbon-bearing materials, battery paste

## Abstract

The recycling of used lead–acid batteries is currently the main source of lead in the world. More than 50% of the weight of a used lead–acid battery is battery paste, in which lead occurs in compounds with oxygen and sulfur. In pyrometallurgical processes of battery paste, coke or coke breeze is used as a traditional additive acting as a fuel/reducer. Due to the constantly high prices of these materials, research is being carried out to find alternative (equally efficient but cheaper) carbon-bearing materials. Those materials can be technological replacements for coke or coke breeze in pyrometallurgical processes. The aim of the presented work was to check the possibility of using fine-grained carbon-bearing materials, in the form of anthracite dust and coal flotation concentrate, for battery paste processing, as replacements for traditionally used coke and coke breeze. As part of the presented work, the following tests were carried out: thermogravimetric tests using battery paste, processes of smelting battery paste in a pit furnace, and processes of smelting battery paste in a rotary furnace. The research results indicate that fine-grained carbon-bearing materials, in the form of anthracite dust and coal flotation concentrate, can be successfully used as a replacement for traditionally used fuels/reducers.

## 1. Introduction

**Figure 1:**
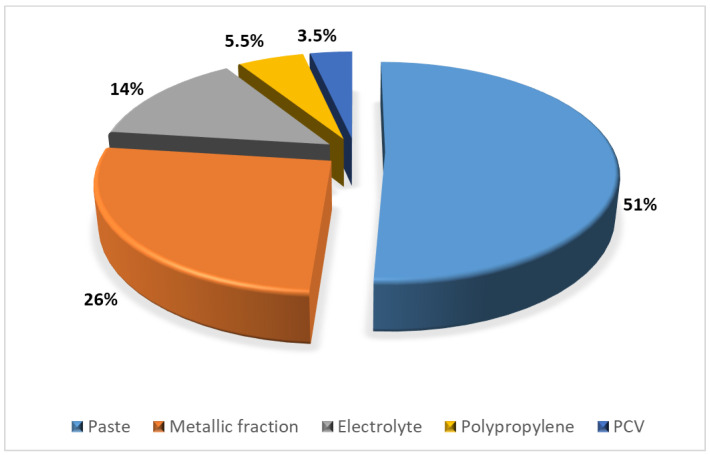
Percentages of materials constituting a lead battery [3].

Scrap from used lead–acid batteries from many types of vehicles, due to its environmental protection and economic aspects, has become the dominant raw material for lead production in the world [1]. The production of 1000 kg of lead from recycled batteries reduces the energy demand by 35–40% compared to the production of the same amount of lead from raw materials [2]. The starting point for processing used batteries is their dismantling and separation into individual fractions. During battery recycling, lead is recovered from the metallic fraction and from the battery paste. The proportions of the different fractions that make up a lead–acid battery are shown in Figure 1. The processing of battery paste, in which lead occurs in compounds with oxygen and sulfur (PbSO_4_, PbO, PbO_2_, PbS), is carried out in pyrometallurgical and hydrometallurgical processes. In Poland, pyrometallurgical processes dominate.

The pyrometallurgical processing of battery paste allows for the recovery of the lead contained in it as a result of exposure to high temperature and suitable reducing materials. The lead content of the paste usually exceeds 70%. The process can be carried out in several types of aggregates, depending on the technology used at a given plant. Among the most important of these are the following:

- Shaft processing;
- Rotary kiln processing;
- Engitec Technologies’ CX integrated processing;
- Isasmelt processing.

As feedstocks for the shaft furnace smelting process, the following additives are used in addition to the battery paste itself [4,5,6,7,8,9]:

- Coke—as an energy source and reducing material;
- Iron—to bind sulfur;
- Fluxes—CaO, SiO_2_, and Na_2_CO_3_.

During the processing of battery paste in a shaft furnace, at a temperature of about 1200 °C, the following chemical reactions take place, among others [4,6,10]:

Carbon monoxide formation:

C + O_2_ = CO_2_ (1)

C + ½ O_2_ = CO (2)

C + CO_2_ = 2 CO (3)

Direct reduction reactions:

2 PbO + C = 2 Pb + CO_2_ (4)

PbSO_4_ + 2 C = PbS + 2 CO_2_ (5)

2 PbSO_4_ + Na_2_CO_3_ + Fe + 9 C = 2 Pb + FeS·Na_2_S + 9 CO + CO_2_ (6)

Indirect reduction reaction:

PbO + CO = Pb + CO_2_ (7)

Reaction between lead sulfide and iron:

PbS + Fe = Pb + FeS(8)

The product obtained from this processing of battery paste is crude lead, which is then further refined.

In the case of processing battery paste in a rotary kiln (analogous to the shaft process), coke, iron, and slag-forming components (Na_2_CO_3_, SiO_2_) are introduced as input additives. The energy required for the process is obtained by a coal combustion reaction and by using an additional energy source in the form of an oil burner.

During the smelting of battery paste in a rotary furnace, we are dealing with analogous reactions to those in the shaft furnace (reactions 1–8) and, additionally, with the following processes [9,11,12,13]:

- The reduction of PbO_2_ to metallic lead;
- The transition of PbS to metallic lead.

PbS + 2 PbO = 3 Pb + SO_2_ (9)

PbS + PbO_2_ = 2 Pb + SO_2_ (10)

In the integrated CX process, preliminary desulfurization of the battery paste with Na_2_CO_3_ is carried out before the actual smelting process in the rotary kiln. The product of this process is desulfurized paste and crystalline sodium sulfate [3,14,15]. The battery paste is also desulfurized before smelting in the Isasmelt furnace, which is a shaft furnace equipped with a lance that is lowered from the top, through which air and fuel are fed during the process [16,17].

As already mentioned, pyrometallurgical processes belong to the group of technologies that traditionally use coke or coke breeze as a feedstock additive. On the one hand, their role is to provide (as a result of combustion reactions) the heat needed to heat the feedstock and, thus, for the desired chemical reactions to take place in the metallurgical unit. On the other hand, they participate in direct or indirect reduction reactions of metal compounds, which are the main metal-bearing components of the feedstock. Due to the constantly high prices of these fuels/reducers, research is being carried out with a view to finding alternative (equally efficient but cheaper compared to coke and coke breeze) carbon-bearing materials that can be their technological substitutes. The few works on this subject concern processes for obtaining copper, iron, and zinc [18,19,20,21,22].

The purpose of the presented work was to test the feasibility of using fine coal-bearing materials, in the form of anthracite dust and coal flotoconcentrate, for the processing of battery paste, as replacements for the traditionally used coke and coke breeze. The fine-grained carbon-bearing materials selected for this research are raw materials commercially available on the market.

As part of the presented work, the following studies were carried out:

- Thermogravimetric studies using battery paste;
- Smelting processes of battery paste in a pit furnace;
- Smelting processes of battery paste in a rotary kiln.

## 2. Materials and Methods

In the conducted tests, battery paste was used, the chemical composition of which is given in Table 1 (data provided by the battery scrap processing plant from which the paste was obtained). Fine-grained carbon-bearing materials in the form of anthracite dust and coal flotoconcentrate were used as alternative reducers. As technological additives, Fe (grain size 45–150 µm) and Na_2_CO_3_ (pure for analysis) were used, in analogy with industrial conditions. For comparison, tests were also carried out using a traditional reductant, i.e., coke. The basic parameters of the reducers used are presented in Table 2 (data provided by mining companies producing specific coal-bearing materials).

The primary instrument used in the first stages of this work was a thermal analyzer from Netzsch (Selb, Germany), model STA 449 F3 Jupiter.

Before the experiment, a sample of a certain mass (about 150 mg) was placed in a DTA/TG (Differential Thermal Analysis/Thermogravimetric Analysis) crucible made of Al_2_O_3_ and then attached to a measuring head in the analyzer’s working chamber.

All measurements were carried out in an air atmosphere. The adopted sample heating program consisted of three main steps:

- Heat up the sample at a rate of 20 °C/min to the selected temperature;
- Maintain such temperature for a period of 120 min;
- Cool down the sample to 700 °C at a rate of 20 °C/min.

Temperatures of 1000 and 1200 °C were selected as the isothermal holding temperatures of the samples during the TG analysis. A 10% addition of carbon-bearing material was used. In order to determine the effect of the form of the material introduced into the thermal analyzer on the speed of the reduction process, this study included carrying out measurements for the material both in bulk form and subjected to agglomeration.

The second stage of the research involved smelting processes of lead from battery paste carried out in a resistance pit furnace. The chemical composition of the prepared mixtures and the basic parameters of the smelting process are given in Table 3. The tests were carried out for material introduced into the furnace both in bulk form (ingredients mixed together in loose form) and in agglomerate form (materials after the lumping process, with a diameter of 0.5 to 1.5 cm). The melts were carried out in crucibles made of Al_2_O_3_. The single mass of feedstock, in both bulk and granular forms, placed in the crucible was 400 g (the lead content in the sample was 302.4 g). As input additives, like those used in industry, sodium carbonate (in amounts of 5 and 10% by weight of the paste) and iron powder (in the amount of 10% by weight of the paste) were used.

**Figure 2:**
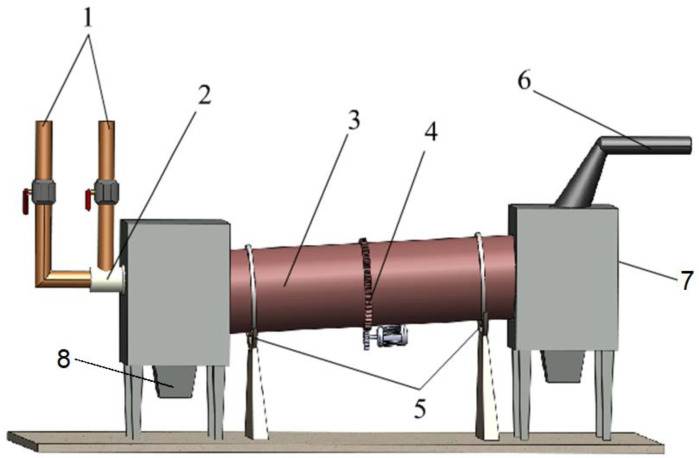
Schematic of the rotary kiln used in this study: 1—gas and air supply; 2—burner; 3—rotary kiln; 4—kiln drive system; 5—rollers; 6—gas discharge; 7—charging; 8—metal discharge.

In the third stage of the study, a rotary kiln was used. A diagram is shown in Figure 2. The use of a rotary furnace in this study made it possible to verify the results obtained during the previous stages of the research. The rotary furnace was a device with a design similar to that of the units commonly used on an industrial scale for smelting lead from battery paste. This furnace was fueled by a burner, to which a mixture of air and natural gas was supplied in a ratio of 5:1. This allowed us to achieve an operating temperature in the range of 1200–1250 °C. The unit could operate continuously, and the use of a rotating furnace working chamber facilitated the mixing of the feedstock and the renewal of its contact surface with the furnace atmosphere. The design of the furnace also allowed for changing the angle of the working chamber, which had a direct bearing on the residence time of the feedstock. Material in granulated form (analogous to that used in industrial processes) was used as the feedstock for the aggregate. For each of the smelting operations carried out in the rotary kiln, a granulated charge of 15 kg was prepared.

The lead smelting process in the metallurgical unit consisted of the following steps:

- Igniting the gas burner and heating the furnace to the desired operating temperature (1200 °C).
- Setting the rotation of the furnace chamber to 1 rpm.
- Setting the angle of the oven to 5°.
- Charging the material in batches in the upper part of the furnace. As each charge moved into the interior of the furnace, another charge was added.
- Passage of the material at a rate of 5 kg/h through the furnace chamber (the feed was given in portions of approx. 5 kg, and the transition time was 1 h).
- Collecting the products of the battery paste reduction process.

The metallic fractions and slags obtained as a result of the reduction processes were subjected to chemical analysis. The concentrations of the elements after microwave digestion were determined using the inductively coupled plasma atomic emission spectrometry (ICP-AES) method. A Varian 710-ES spectrometer (Palo Alto, CA, USA) equipped with a glass SeaSpray nebulizer and a double-pass glass cyclonic spray chamber were applied.

## 3. Results and Discussion

**Figure 3:**
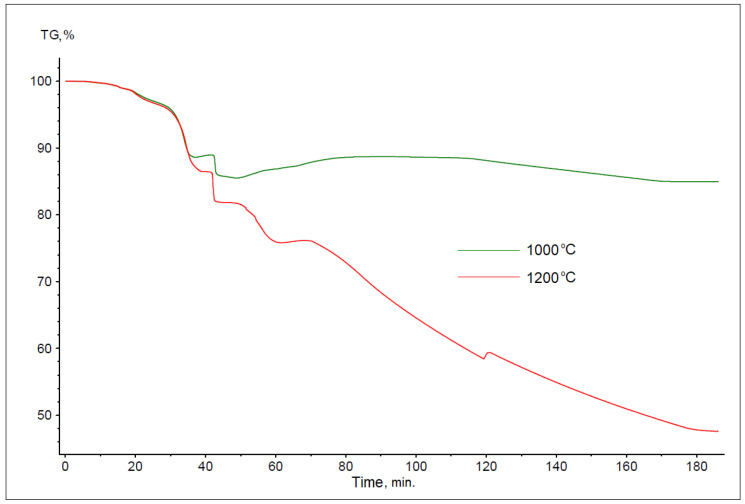
Summary of thermogravimetric reaction curves for battery paste, anthracite dust, and Fe at temperatures of 1000 and 1200 °C (bulk samples).

The thermogravimetric curves obtained during the reduction of battery paste using anthracite dust are shown in Figure 3. The shape of these curves varies depending on the process temperature, with an increase in the process temperature resulting in a significant increase in sample weight loss. The total mass losses in the samples tested were 14.99% for 1000 °C and 52.42% for 1200 °C.

**Figure 4:**
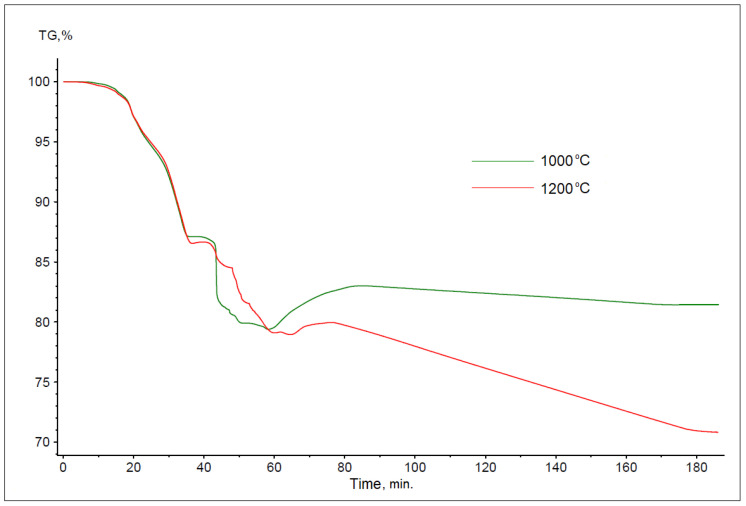
Summary of thermogravimetric reaction curves for battery paste, flotoconcentrate, and Fe at temperatures of 1000 and 1200 °C (bulk samples).

The thermogravimetric curves obtained for the reduction process of battery paste using flotoconcentrate are shown in Figure 4. As with anthracite dust, the course of the TG curves varies depending on the process temperature. The determined total weight losses of the samples were 18.59% for a temperature of 1000 °C and 29.21% for a temperature of 1200 °C.

**Figure 5:**
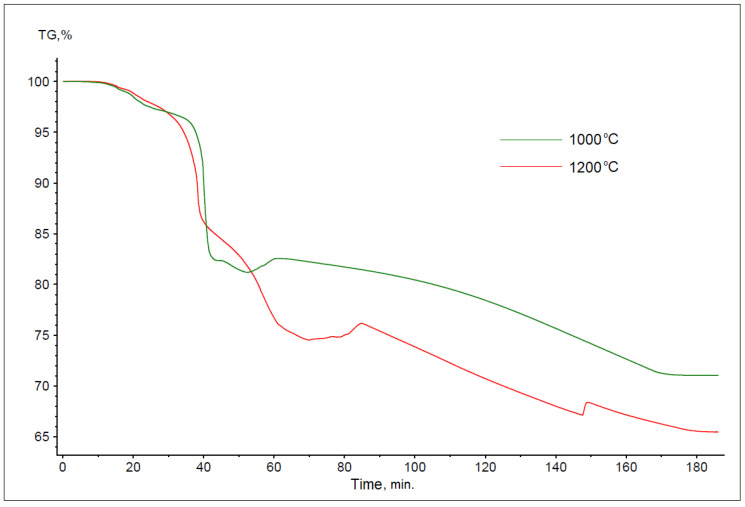
Summary of thermogravimetric curves of the reactions between battery paste, coke, and Fe at temperatures of 1000 and 1200 °C (bulk samples).

On the other hand, Figure 5 shows the curves obtained for the reduction process of battery paste using coke. As before, it was found that an increase in temperature increased the weight loss of the sample, with it reaching 28.96% at 1000 °C, while at 1200 °C, it was 31.67%.

In comparing the kinetics of the reaction curves between the selected carbon-bearing materials and the battery paste, it can be stated that the rate of mass loss (and, thus, reaction) in the case of coke is clearly lower than that in the case of the other two reducers. This may indicate their greater reduction capacity or a higher content of volatile parts in the materials tested.

**Figure 6:**
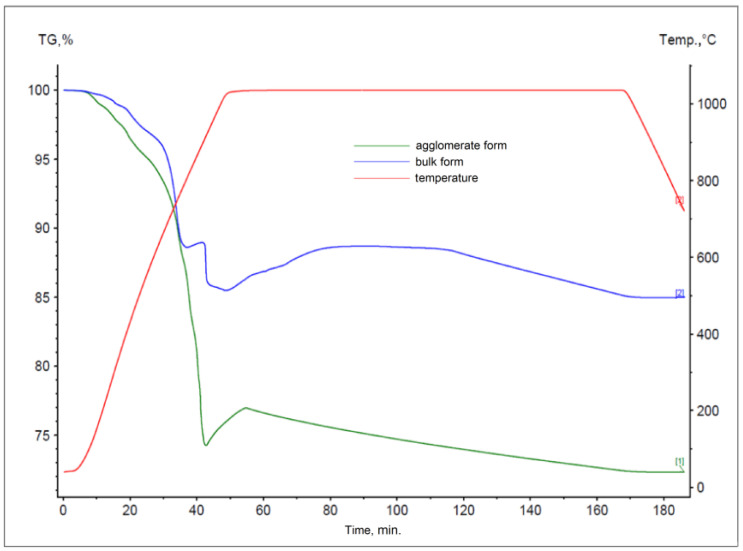
Comparison of thermogravimetric curves of reactions between battery paste, anthracite dust, and Fe at 1000 °C for bulk and agglomerate samples.

**Figure 7:**
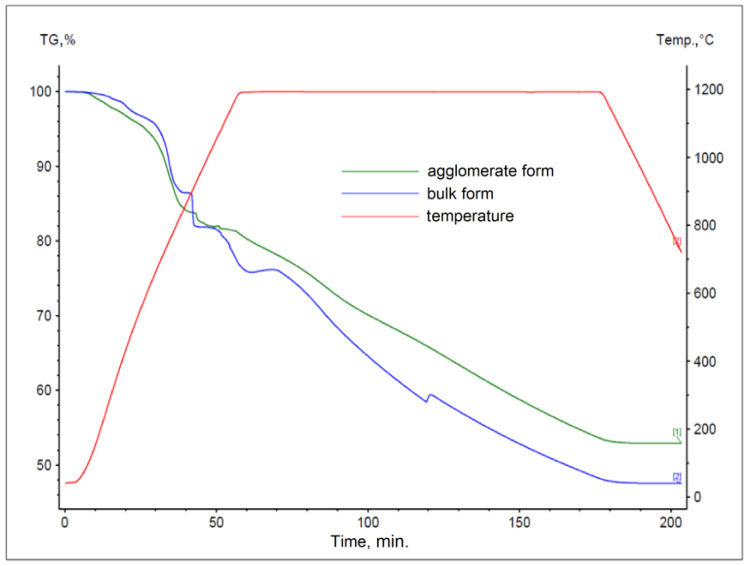
Comparison of thermogravimetric curves of reactions between battery paste, anthracite dust, and Fe at 1200 °C for samples in bulk and agglomerate form.

**Figure 8:**
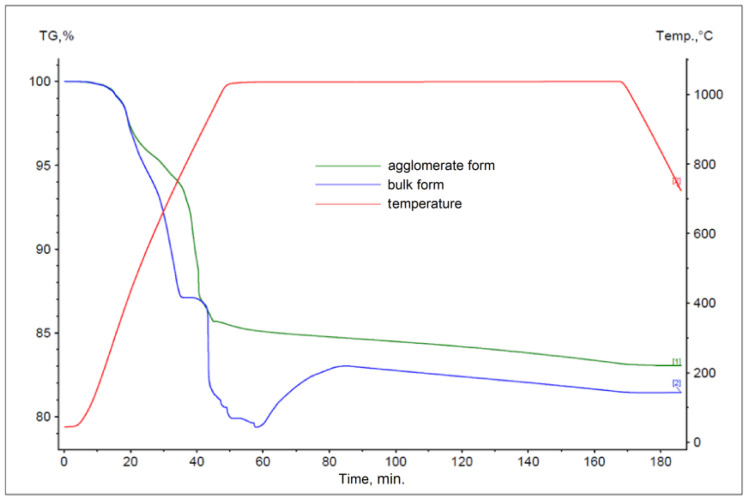
Comparison of thermogravimetric curves of reactions between battery paste, flotoconcentrate, and Fe at 1000 °C for bulk and agglomerate samples.

**Figure 9:**
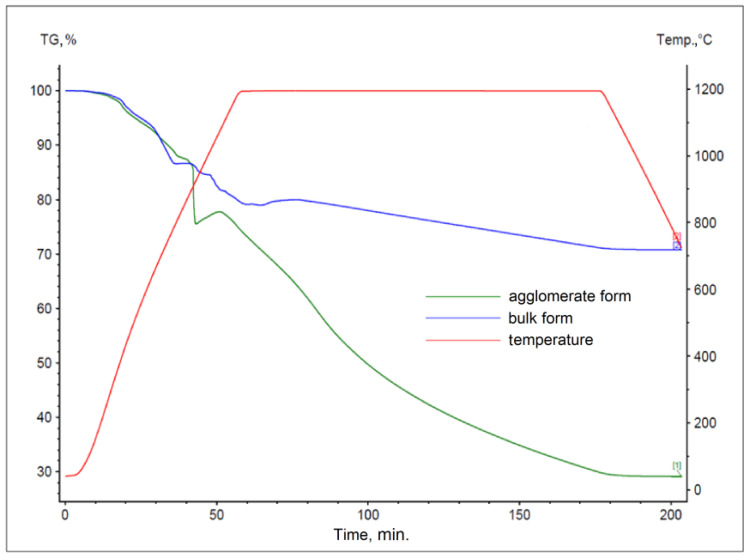
Comparison of thermogravimetric curves of reactions between battery paste, flotoconcentrate, and Fe at 1200 °C for bulk and agglomerate samples.

**Figure 10:**
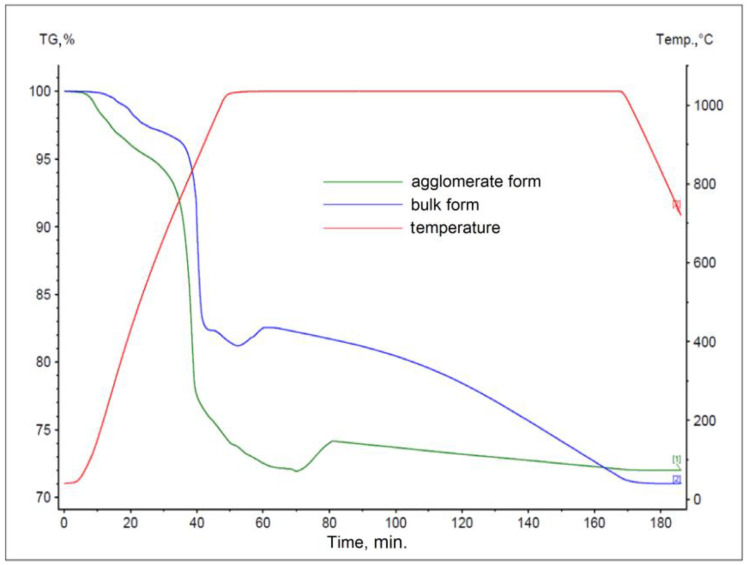
Comparison of thermogravimetric curves of reactions between battery paste, coke, and Fe at 1000 °C for bulk and agglomerate samples.

**Figure 11:**
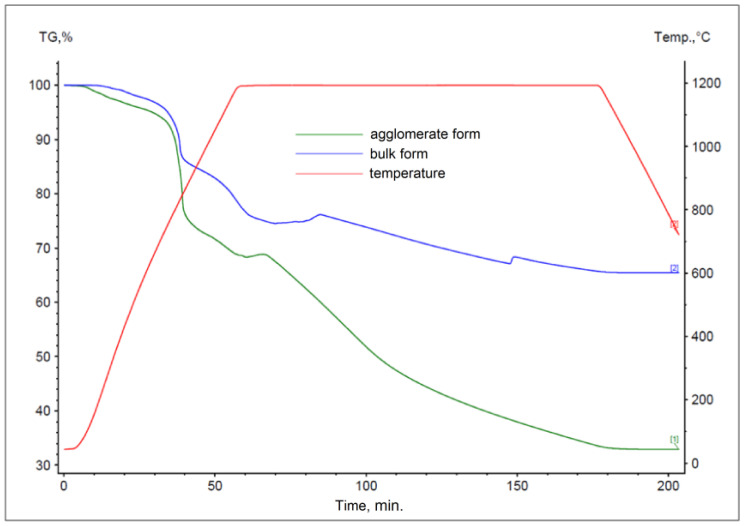
Comparison of thermogravimetric curves of reactions between battery paste, coke, and Fe at 1200 °C for bulk and agglomerate samples.

The results obtained for materials subjected to the agglomeration process are shown in Figure 6, Figure 7, Figure 8, Figure 9, Figure 10 and Figure 11, with the curves obtained for materials in bulk form also plotted for comparison. By analyzing the recorded thermogravimetric curves, it was found that in the case of samples containing anthracite dust and coke as carbon-bearing materials, there was a clear effect of the form in which the material was introduced for testing. The use of material in the form of agglomerate affected the weight loss of the samples, regardless of the process temperature. For samples where flotoconcentrate was used as the carbon-bearing material, an analogous effect occurred for the sample at 1200 °C. For the process taking place at a lower temperature, there was no clear relationship between sample weight loss and the form of material introduced into the process.

The results of the study of the smelting process of battery paste in a resistance pit furnace are summarized in Table 4, Table 5 and Table 6. From an analysis of the smelting processes of battery paste with the carbon-bearing materials used, it can be concluded that an increase in the content of reductant in the batch mixture does not increase the metallic lead yield. Based on the results of this study, it can be concluded that calcium carbonate does not play an important role during the smelting of battery paste. Regarding the effect of the form of the batch mix (loose or granular form), it was found that the use of a granular mix markedly reduced the metal yield. This was especially visible when coke was used as a reducing agent.

When comparing the metal yields for processes carried out using individual carbon-bearing materials, we noted that the highest yield values were characterized by samples wherein flotoconcentrate was used. Chemical analyses of selected post-process slags (from samples characterized by the highest metal yield), the results of which are summarized in Table 7, showed that the lowest lead content was characterized by the slag from sample No. 20, wherein flotoconcentrate was used as a carbon-bearing material. In the case of the other two coal-bearing materials (anthracite dust and coke), the lead content of the waste slag was at a higher level, at 3.2 wt.% and 5.4 wt.%, respectively.

In order to determine the quality of the obtained metal, an analysis of its chemical composition was carried out. The results are shown in Table 8. The obtained lead was characterized by high purity, i.e., above 99 wt.%. Pb.

Table 9 shows the characteristics of the feedstock materials and summarizes the results of the battery paste smelting process in the rotary kiln. In all cases, the determined metal yields were similar and amounted to about 80%, which indicates the similar ability of all carbon-bearing materials to reduce battery paste. The metallic phase obtained as a result of the processes in all samples studied was characterized by a high lead content exceeding 99 wt.% (Table 10). The slag formed as a result of smelting the battery paste was characterized (relative to that from smelting in the resistance pit furnace) by a higher lead content (Table 11).

The presented research results show the possibility of using the tested carbon-bearing materials in metallurgical processes. The prices of the individual materials also support this, as both anthracite dust (120 USD/ton) and flotation concentrate (75 USD/ton) are much cheaper than the commonly used coke (210 USD/ton).

## 4. Conclusions

The nature of thermogravimetric curves for the reduction process of battery paste does not change as a function of temperature. This is due to the fact that in this particular case, an increase in temperature does not affect the type of chemical reactions occurring in the sample. However, an increase in temperature has a significant effect on the speed and kinetics of the chemical reactions taking place, which translates into an increase in the loss of sample mass and, thus, the metal yield.

The smelting processes of battery paste using anthracite dust, flotoconcentrate, and, for comparison, coke, carried out in a resistance pit furnace and a rotary kiln, showed no effect of the reductant used on the purity of the crude lead obtained. The use of flotation concentrate and anthracite dust as reducing materials for the smelting of battery paste in a rotary furnace resulted in a slight (ca. 2%) reduction in metal yield in relation to that obtained using coke breeze. Therefore, it can be assumed that the proposed fine-grained carbon-bearing materials can be technological substitutes for coke in industrial battery paste processing.

The metal masses obtained for the battery paste reduction tests in the rotary kiln using the proposed alternative carbon-bearing materials (6.47 kg for flotoconcentrate and 6.41 for anthracite dust) did not differ from the value obtained for the coke traditionally used in metallurgy (6.65 kg).

In conclusion, it can be said that the results obtained from this study on the reduction of battery paste show the possibility of using both flotoconcentrate and anthracite dust as substitutes for the coke that is commonly used in industrial conditions.

## Figures and Tables

**Table 1:** Chemical composition of battery paste.

Element	Content, wt.%	Element	Content, wt.%
Pb	75.6	C	2.11
Cd	0.003	S	3.65
Fe	0.45	Sb	0.17
Hg	0.1 ppm	Zn	0.020
Mn	0.010	H_2_O	8.7

**Table 2:** Selected quality parameters of carbon-bearing materials used in the research.

Research Material	Sulfur Content, wt.%	Calorific Value, kJ/kg.	Ash Content, % by Mass	Grain Size, mm
Anthracite dust	1.51	27,875	10.7	<0.5
Flotoconcentrate	0.45	20,824	8.71	0.5–2
Coke	0.54	30,000	11.5	1–3

**Table 3:** Experimental conditions and smelting parameters.

Process Parameter	Minimum Value	Maximum Value
Process temperature, °C	1200	
Reducer addition, %mas.	8	12
Batch additives, %mas.	5	10
Smelting time, h	3	

**Table 4:** Summary of test results of smelting of battery paste with anthracite dust addition.

Sample No.	Ingredient Content in the Charge, % by Weight			Form of the Feedstock	Weight of Melted Metal, g	Yield, % by Weight
	Reducer	Fe	Na_2_CO_3_			
1	8	10	5	B	279.7	92.5
2	10			B	254.6	84.2
3	12			B	216.5	71.6
4	8			G	288.5	95.4
5	10			G	231.9	76.7
6	12			G	182.3	60.3
7	8		10	B	295.1	97.6
8	10			B	226.5	74.9
9	12			B	180.8	59.8
10	8			G	300.3	99.3
11	10			G	271.3	89.7
12	12			G	168.4	55.7

B—bulk material, G—material in the form of granules.

**Table 5:** Summary of test results of smelting of battery paste with flotoconcentrate addition.

Sample No.	Ingredient Content in the Charge, % by Weight			Form of the Feedstock	Weight of Melted Metal, g	Yield, % by Weight
	Reducer	Fe	Na_2_CO_3_			
13	8	10	5	B	296.7	98.1
14	10			B	252.8	83.6
15	12			B	179.0	59.2
16	8			G	300.6	99.4
17	10			G	271.3	89.7
18	12			G	202.0	66.8
19	8		10	B	288.5	95.4
20	10			B	299.4	99.0
21	12			B	248.0	82.0
22	8			G	300.0	99.2
23	10			G	277.3	91.7
24	12			G	163.0	53.9

B—bulk material, G—material in the form of granules.

**Table 6:** Summary of test results of smelting of battery paste with coke addition.

Sample No.	Ingredient Content in the Charge, % by Weight			Form of the Feedstock	Weight of Melted Metal, g	Yield, % by Weight
	Reducer	Fe	Na_2_CO_3_			
25	8	10	5	B	287.3	95.0
26	10			B	285.2	94.3
27	12			B	288.8	95.5
28	8			G	261.0	86.3
29	10			G	255.5	84.5
30	12			G	250.4	82.8
31	8		10	B	297.9	98.5
32	10			B	259.2	85.7
33	12			B	254.9	84.3
34	8			G	295.7	97.8
35	10			G	280.9	92.9
36	12			G	230.1	76.1

B—bulk material, G—material in the form of granules.

**Table 7:** Lead content in post-process slag from smelting of battery paste in a resistance pit furnace.

Sample No.	Lead Content, wt.%
10	3.24
20	1.93
31	5.38

**Table 8:** The chemical composition of the metallic phase obtained in the smelting of battery paste in a resistance pit furnace.

Sample No.	Content, wt.%				
	Pb	Fe	Zn	Sb	Cu
10	99.29	0.23	<0.0010	0.08	0.39
20	99.08	0.45	<0.0015	0.02	0.44
31	99.29	0.23	-	0.12	0.35

**Table 9:** Summary of results of smelting of battery paste in a rotary kiln.

Sample No.	Type of Carbon-Bearing Material	Weight of Lead Contained in the Charge, kg	Ingredient Content in the Charge, wt.%			Weight of Smelted Metal, kg	Yield, %
			Reducer	Fe	Na_2_CO_3_		
1	A	8.05	10	10	5	6.41	79.6
2	F					6.47	80.3
3	C					6.65	82.5

A—anthracite dust, F—flotoconcentrate, C—coke.

**Table 10:** Chemical composition of the metallic phase obtained in the smelting of battery paste in a rotary kiln.

Carbon-Bearing Material	Content, wt.%				
	Pb	Fe	Zn	Sb	Cu
Anthracite dust	99.86	0.04	-	0.02	0.07
Flotoconcentrate	99.91	0.01	-	0.02	0.05
Coke	99.90	0.01	-	0.02	0.06

**Table 11:** Lead content in post-process slag from smelting of battery paste in a rotary kiln.

Carbon-Bearing Material	Lead Content, wt.%
Anthracite dust	5.79
Flotoconcentrate	7.24
Coke	5.41

## Data Availability

The original contributions presented in this study are included in the article. Further inquiries can be directed to the corresponding author.
